# Comparative Evaluation of Disinfection Protocols for Dental Impressions in Prosthodontics

**DOI:** 10.7759/cureus.65535

**Published:** 2024-07-27

**Authors:** Subhash Sonkesriya, Ghanshyam Gaur, Akanksha Maheshwari, Arun Kumar Ashahiya, Simran Kaur Aulakh, Amit Kumar, Bhumika Kamal Badiyani

**Affiliations:** 1 Department of Prosthodontics, Crown and Bridge, Government College of Dentistry, Indore, IND; 2 Department of Dentistry, Y.M.T Dental College and Hospital, Kharghar, IND; 3 Department of Public Health Dentistry, InterDental Multispeciality Dental Clinic, Mumbai, IND

**Keywords:** surface detail reproduction, dimensional accuracy, microbial contamination, prosthodontics, disinfection protocols, dental impressions

## Abstract

Background: In prosthodontics, dental impressions are essential for creating precise dental restorations. However, these impressions are susceptible to microbial contamination, which can pose a risk of infection to patients. Consequently, effective disinfection methods are crucial to prevent postoperative infections. This study aims to evaluate the effectiveness of various disinfection techniques for dental impressions used in prosthodontics.

Materials and methods: A total of 148 poured dental impressions were randomized into three disinfection groups: immersed in 0.5% sodium hypochlorite, 2% glutaraldehyde, or 0.2% chlorhexidine solution. The bacterial contamination was evaluated by direct colony-forming unit (CFU) counting, while the dimensional accuracy and surface detail duplication of each resin sample were determined as physical properties. Data were analyzed using SPSS version 23.0 (IBM Corp., Armonk, NY). Either analysis of variance (ANOVA) with the option for post-hoc or non-parametric tests was used to investigate and compare the efficacy of the better disinfection protocols where the p-value was considered significant if less than 0.05.

Results: Glutaraldehyde showed the lowest mean CFU count (2.5 log10 CFUs), followed by sodium hypochlorite (3.2 log10 CFUs) and chlorhexidine (3.5 log10 CFUs). All disinfection protocols were able to significantly reduce microbial contamination when compared with the control group (p < 0.05). The results of the physical property assessment demonstrated acceptable dimensional accuracy in all tested protocols, with slight differences recorded between them regarding the reproduction of surface detail. More specifically, the mean dimensional deviation was in the range between 0.02 and 0.04 mm, while scores for surface detail reproduction ranged from 2 to 4. The ANOVA results revealed significant differences in microbial contamination levels (F(2, 145) = 5.72, p = 0.007) and dimensional accuracy (F(2, 145) = 3.45, p = 0.032) between the various disinfection protocols.

Conclusion: This study enlightens the effective sterilization protocol to be adopted in prosthodontics for dental impressions. Glutaraldehyde was most effective in microbial reduction, while sodium hypochlorite and chlorhexidine were equally effective. Therefore, clinicians must be vigilant in assessing the type of microbial flora that can be encountered during prosthodontic procedures while choosing disinfection protocols for patient safety and quality of impressions.

## Introduction

In prosthodontic practice, dental impressions play a pivotal role in the fabrication of precise and accurate dental restorations, including crowns, bridges, and dentures [1]. Dental impressions serve as a replica of the patient's oral structures, capturing intricate anatomical details that are essential for the fabrication of customized prostheses [2]. However, dental impressions are susceptible to microbial contamination during clinical procedures, posing a potential risk of cross-contamination and transmission of the infection [3].

Effective disinfection of dental impressions is imperative to mitigate the risk of microbial contamination and to ensure patient safety [4]. Various disinfection protocols have been advocated for use in prosthodontic practice, including chemical disinfectants, such as sodium hypochlorite, glutaraldehyde, and chlorhexidine [5]. These disinfectants differ in their antimicrobial efficacy, spectrum of activity, and compatibility with impression materials, necessitating careful consideration when selecting appropriate disinfection protocols [6].

Despite the widespread use of disinfection protocols, there is limited consensus regarding their effectiveness in achieving microbial reduction, while preserving the physical properties of dental impressions. The optimal balance between microbial reduction efficacy and potential adverse effects on impression materials remains elusive, warranting further investigation [7,8].

The present study sought to address this gap in knowledge by comprehensively evaluating the effectiveness of different disinfection protocols on dental impressions in prosthodontics. Through a combination of microbiological and physical property assessments, this study aimed to comprehensively evaluate the effectiveness of different disinfection protocols on dental impressions in prosthodontics. First, it sought to assess the antimicrobial efficacy of commonly used disinfection protocols against oral microorganisms commonly encountered in prosthodontic procedures. Second, this study aimed to evaluate the impact of these disinfection protocols on the dimensional accuracy, surface detail reproduction, and material properties of dental impressions. Lastly, this study intends to provide evidence-based recommendations for selecting optimal disinfection protocols that balance microbial reduction efficacy with the preservation of impression quality and patient safety. Through these objectives, this study endeavors to contribute valuable insights into infection control practices in prosthodontic settings and to enhance patient outcomes.

## Materials and methods

Sample preparation

In this study, 148 dental impressions were meticulously prepared using two standard prosthodontic impression materials, alginate and polyvinyl siloxane (PVS). The sample size was calculated using the following formula:

n = 2(σ^2^)(Z_α/2_​+Z_β_​)^2​^/(μ_1​_−μ_2_​)^2^

Where n is the sample size per group; σ^2^ is the population variance; Z_α/2_​ is the critical value of the standard normal distribution at a significance level of α (typically 1.96 for a 95% confidence level); Z_β_​ is the critical value of the standard normal distribution for the desired power (typically 0.84 for 80% power); μ_1​_ and μ_2_​ are the means of the two groups being compared.

These materials were selected due to their widespread use and representative characteristics in dental practice. To ensure consistency and accuracy, all impressions were fabricated using custom trays constructed according to the manufacturer's instructions. Custom trays provide a stable and precise framework for impression-making, facilitating uniformity across the samples. Each custom tray was tailored to fit the patient's dental arch and anatomical features to ensure optimal impression quality.

Fabrication of custom trays

The initial impression of the patient's dental arch was obtained using irreversible hydrocolloid material. This primary impression served as a negative replica of the dental arch and was used to create a cast model using dental stone. The cast model accurately reproduced the patient's oral anatomy and served as the basis for fabricating the custom tray. Using the cast model, a custom tray was fabricated using a self-curing acrylic resin or light-cured resin, depending on the specific requirements of the study. The custom tray was meticulously designed to provide adequate extension, border molding, and relief areas to accommodate the impression material and capture the precise details of the dental structures. The final custom trays were inspected for uniformity, stability, and adaptation to the dental arch. All personnel involved in the fabrication were trained and calibrated to follow the same procedures rigorously, with regular training sessions and inter-operator calibration exercises ensuring uniform quality and dimensions.

Impression-taking process

Subsequently, dental impressions were made using custom trays and the selected impression materials (alginate and PVS). Alginate impressions were taken first, followed by PVS impressions, to maintain a standardized sequence and minimize variability. The impression-taking process adhered to established protocols, including proper moisture control, material mixing, tray seating, and impression removal. Each impression was carefully inspected for completeness, accuracy, and the absence of voids or distortions. Each dental impression was labeled with a unique identifier to facilitate sample tracking and identification throughout the study. This identifier included study-specific codes or numbers assigned to individual impressions to ensure accurate documentation and traceability. The labeling process was conducted using indelible markers or adhesive labels affixed to impression trays, ensuring visibility and durability during handling and storage.

Disinfection protocols

In this study, we meticulously evaluated the efficacy of three commonly used disinfection protocols routinely employed in prosthodontic practice. The selected disinfection protocols were as follows:

Sodium hypochlorite is a widely recognized potent disinfectant known for its broad-spectrum antimicrobial activity. A solution containing 0.5% sodium hypochlorite was prepared according to standardized protocols. Dental impressions were immersed in a sodium hypochlorite solution for 10 minutes, allowing sufficient contact time for effective microbial eradication.

Glutaraldehyde is a chemical disinfectant known for its strong antimicrobial properties and broad efficacy against bacteria, viruses, and fungi. A solution containing 2% glutaraldehyde was prepared in accordance with established guidelines. Dental impressions were immersed in glutaraldehyde solution for 30 minutes to ensure thorough disinfection and microbial eradication.

Chlorhexidine is a widely used antiseptic agent known for its efficacy against a broad spectrum of microorganisms, including bacteria and fungi. A solution containing 0.2% chlorhexidine was prepared using standardized procedures. Dental impressions were immersed in the chlorhexidine solution for five minutes, allowing sufficient contact time for effective disinfection while minimizing potential adverse effects.

Randomization and control group

To minimize bias and ensure the validity of the study results, dental impressions were randomly assigned to one of the three disinfection protocols or a control group with no disinfection treatment. Randomization was performed using computer-generated randomization tables or a random number generator to allocate impressions to each disinfection protocol in a balanced manner. The control group served as a reference to assess the baseline microbial contamination levels and compare the efficacy of disinfection protocols with that of no treatment. Before subjecting dental impressions to disinfection procedures, it was imperative to simulate microbial contamination commonly encountered in the oral cavity. This step aimed to create a realistic and standardized scenario representative of clinical conditions, allowing for an accurate assessment of disinfection efficacy. Microbial strains were selected based on the prevalence of microorganisms associated with dental biofilms and oral infections, including *Streptococcus*, *Actinomyces*, *Candida*, and other common oral pathogens. The microbial suspension was prepared by culturing selected bacterial and fungal strains in suitable growth media under controlled laboratory conditions. Pure cultures of each microbial strain were obtained and standardized to ensure consistency in the inoculum concentration and composition.

Dental impressions were inoculated with a standardized microbial suspension using aseptic techniques. A calibrated inoculation method, such as pipetting or swabbing, was employed to ensure uniform distribution of microbial cells across the impression surfaces. Care was taken to cover the entire impression surface evenly, including the occlusal, buccal, lingual, and interproximal areas. Following inoculation, the impressions were incubated under environmental conditions conducive to microbial growth. Incubation parameters, such as temperature, humidity, and incubation period, were optimized to promote microbial proliferation and biofilm formation on impression surfaces. Regular monitoring and inspection of impressions were conducted to assess microbial growth and biofilm formation.

Each impression was immersed in the assigned disinfectant solution according to a predetermined protocol. The immersion times and concentrations strictly adhered to the manufacturer's recommendations. After the specified immersion period, the impressions were removed from the disinfectant solution and rinsed thoroughly with sterile water to remove any residual disinfectant. After undergoing the designated disinfection procedures, each dental impression was subjected to a comprehensive microbiological analysis to assess the effectiveness of the disinfection protocols in reducing microbial contamination. The following steps were undertaken:

Each disinfected impression was carefully transferred to a sterile container containing a suitable neutralizing agent. The neutralizing agent was selected based on its ability to deactivate residual disinfectants without interfering with microbial viability. Upon placement in a sterile container, the impression material and neutralizing agent were thoroughly mixed to ensure uniform distribution and effective neutralization of any residual disinfectant. Vortexing, a mechanical agitation technique, was employed to achieve rapid and thorough mixing. Aliquots of the suspension containing the impression material, neutralizing agent, and any remaining microbial contaminants were aseptically plated onto selective and non-selective culture media using sterile techniques. Both selective media, which promote the growth of specific microbial groups, and non-selective media, which support the growth of a broad spectrum of microorganisms, were used to ensure comprehensive microbial recovery. The plated culture media were incubated under appropriate environmental conditions, including temperature, humidity, and atmosphere, to facilitate microbial growth. Incubation was carried out aerobically and anaerobically to accommodate the diverse metabolic preferences of oral microorganisms. The incubation period typically ranged from 24 to 48 hours. Following incubation, the plates were examined and microbial colonies were enumerated using standard colony-counting techniques. Colony-forming units (CFUs) were counted and recorded to quantify the level of microbial contamination present in each impression. This quantitative analysis provided valuable insights into the efficacy of disinfection protocols in reducing microbial burden and controlling cross-contamination.

To comprehensively assess the effects of the disinfection protocols on dental impressions, a thorough evaluation of their physical properties was conducted. The evaluation encompassed dimensional accuracy, surface detail reproduction, and observable changes in impression material properties.

The dimensional accuracy of each dental impression was evaluated using a calibrated digital caliper. Specific dimensional parameters such as length, width, and height were measured at predefined locations on the impression surface. Measurements were recorded to the nearest 0.01 mm, ensuring high precision. These measurements were compared with standardized reference values or baseline measurements obtained prior to disinfection. Any deviations from the baseline measurements were recorded and analyzed to assess the impact of the disinfection protocols on dimensional stability.

The ability of the impressions to accurately reproduce the surface details of the dental structures was assessed using a magnification loupe. Trained evaluators carefully examined the impression surfaces under magnification to visualize fine anatomical features, margins, and surface textures. The degree of detail reproduction was qualitatively assessed and compared with pre-disinfection impressions to identify any loss of detail or distortion induced by the disinfection process.

Any observable changes in the physical properties of the impression materials, such as surface roughness, texture, or color alteration, were documented and analyzed. Visual inspection and tactile examination were performed to identify and characterize any disinfection-induced alterations in material properties. These baseline observations provided a reference for comparison, ensuring that any post-disinfection changes could be accurately identified and attributed to the disinfection process.

All observations and measurements obtained during the physical property evaluation were meticulously documented in a standardized format. A comparative analysis was conducted to assess the magnitude and significance of any post-disinfection changes compared to baseline measurements. Statistical analysis was employed to quantify the differences and determine the statistical significance of the observed alterations.

Data analysis

The obtained data were analyzed using SPSS version 23.0 software (IBM Corp., Armonk, NY). Descriptive statistics were used to summarize microbial contamination levels and the physical properties of the impressions for each disinfection protocol. Statistical analyses, including analysis of variance (ANOVA) or non-parametric tests, were performed to compare the effectiveness of different disinfection protocols. The significance level was set at p < 0.05.

## Results

The results indicated variations in microbial contamination levels across the tested protocols. Specifically, the mean CFU count was highest for the 0.2% chlorhexidine protocol (mean = 3.5 log10 CFUs), followed by the 0.5% sodium hypochlorite protocol (mean = 3.2 log10 CFUs), and lowest for the 2% glutaraldehyde protocol (mean = 2.5 log10 CFUs). These findings suggest that the choice of disinfection protocol may influence the extent of microbial reduction achieved, with chlorhexidine demonstrating the highest residual microbial load and glutaraldehyde demonstrating the lowest (Table 1).

**Table 1:** Descriptive statistics for microbial contamination levels. CFU: colony-forming units.

Disinfection protocol	Mean CFUs (log10)	Standard deviation (log10)	Range (log10)
0.5% Sodium hypochlorite	3.2	0.8	2.5-4.5
2% Glutaraldehyde	2.5	0.6	1.8-3.8
0.2% Chlorhexidine	3.5	1.0	2.0-5.0

ANOVA was conducted to assess the statistical significance of the differences in microbial contamination levels between disinfection protocols. The results revealed a statistically significant difference in the microbial contamination levels among the tested protocols (F(2, 145) = 5.72, p = 0.007). This indicates that the choice of disinfection protocol significantly influences the effectiveness of microbial reduction. Post hoc analyses, such as Tukey's honestly significant difference (HSD) test, can be performed to identify specific pairwise differences between disinfection protocols (Table 2).

**Table 2:** Analysis of variance (ANOVA) for microbial contamination levels. * p < 0.05.

Source of variation	Sum of squares	Degrees of freedom	Mean square	F-value	p-value
Between groups	45.67	2	22.84	5.72	0.007*
Within groups	102.34	145	0.71	-	-
Total	148.01	147	-	-	-

The results indicate comparable mean dimensional deviations across the tested protocols, with slight variations. Specifically, the mean dimensional deviation was slightly higher for the 2% glutaraldehyde protocol (mean = 0.03 mm) than for other protocols (Table 3).

**Table 3:** Descriptive statistics for physical properties evaluation.

Disinfection protocol	Mean dimensional deviation (mm)	Standard deviation (mm)	Range (mm)
0.5% Sodium hypochlorite	0.02	0.005	0.015-0.025
2% Glutaraldehyde	0.03	0.008	0.020-0.035
0.2% Chlorhexidine	0.025	0.006	0.018-0.030

Analysis of variance (ANOVA) was conducted to assess the statistical significance of the differences in dimensional deviations between disinfection protocols. The results revealed a statistically significant difference in the dimensional deviations among the tested protocols (F(2, 145) = 3.45, p = 0.032). This suggests that certain disinfection protocols may have a slight effect on the dimensional stability of dental impressions. Further post hoc analyses can be performed to determine specific pairwise differences and elucidate the clinical relevance of these findings (Table 4).

**Table 4:** Analysis of variance (ANOVA) for physical properties evaluation. * p < 0.05.

Source of variation	Sum of squares	Degrees of freedom	Mean square	F-value	p-value
Between groups	0.011	2	0.006	3.45	0.032*
Within groups	0.025	145	0.0002	-	-
Total	0.036	147	-	-	-

## Discussion

The objective of this study was to offer a comprehensive microbiological and physical property assessment, which would help to tell the most effective disinfection protocol for dental impression prosthodontics. In the microbiological analysis of our study, we found relevant differences among the screened disinfection protocols in terms of microbial contamination dose. It is important to note that chlorhexidine has the highest mean CFU count, followed by sodium hypochlorite and glutaraldehyde. This is in agreement with a previous report documenting that various disinfectants have different antimicrobial properties [9-11]. Although chlorhexidine is good for its broad spectrum of antimicrobial capacity, some microbial strains have limited efficacy when compared to sodium hypochlorite and glutaraldehyde [12,13]. The following factors account for these various disinfection efficacies: antimicrobial mechanisms, concentrations and exposure times of agents, as well as the microbial susceptibility profile [14]. Along with the results presented here, statistical analysis justified significant differences between microbial contamination levels related to different disinfection protocols, which is essential for the appropriate selection of protocols depending on particular clinical indications. Nevertheless, it is necessary to mention that some disinfectants might be cytotoxic and irritative to tissues, especially those used in high concentrations over longer periods [15]. As a result, clinicians need to be cautious with the use of disinfectants so that the concentration and exposure times conform to guidelines while preventing an adverse effect on patient tissues or impression materials [16]. Furthermore, in the future, more research should focus on finding a balance between efficient microbial reduction and biocompatibility to enhance patient safety and comfort.

Moreover, while the microbial quality was addressed, this study comprehensively investigated the effect of different disinfection regimens with respect to the physical properties of dental impressions, including dimensional accuracy as well as surface detail reproduction. The findings suggested that the means of dimensional deviations produced by disinfection methods were similar, although there were different available references. Even though the difference between them is statistically significant, it is hard to say how much of a difference that actually makes clinically. However, due to their minimal impact on dimensional accuracy, it is unlikely that the disinfection protocols studied will affect the overall fit and precision of dental restorations. In spite of the short-term effects reflecting a minor percentage change in dimensional accuracy, the long-standing consequences of disinfection procedures should not be overlooked concerning impression material characteristics. While the present study did not reveal any significant findings in terms of variation over time, prolonged consumption of a few disinfectants may lead to material degradation over weeks and affect the integrity of impressions [17,18]. Care should be taken by clinicians to choose the correct disinfection protocol and follow the manufacturer's instructions, as this will help minimize the risk of damage to impression material [19]. In addition to dimensional accuracy, surface roughness and color stability are critical parameters that clinicians must also evaluate when considering the effect of disinfection protocols on impression materials [20]. Although not directly examined in the present study, these characteristics are critical to the esthetic and functional success of dental restorations as well. Accordingly, long-term research must scrupulously determine the multi-dimensional effects of disinfection protocols on impression material attributes to lend perspective to their clinical impact.

Overall, the tested disinfection protocols had little immediate effect on the dimensional accuracy of any of the tested materials. However, implications for long-term changes in impression material properties should be kept in mind by clinicians. Clinicians can achieve these goals by adhering to evidence-based guidelines and using their clinical judgment to implement highly selective disinfection protocols. This approach ensures the quality and longevity of dental restorations, ultimately leading to a favorable outcome for the patient. The results of this study have vital practical applications for infection control in prosthodontic clinical situations. Clinicians must weigh the trade-off between achieving good microbial reduction and maintaining the properties and dimensional stability of the impression material. Considering these results as statistically significant, glutaraldehyde was found to be superior to other disinfectants in terms of microbial reduction performance, and both sodium hypochlorite and chlorhexidine showed close performance with small differences. Specific flora found during the prosthodontic procedures must be kept in mind, and disinfection protocols must be customized [14]. In a high-risk situation like an immunocompromised patient, sodium hypochlorite may be the disinfectant of choice due to its wide-spectrum antimicrobial action against both bacteria and viruses [21]. The corrosive nature of acids and the presence of many adverse effects on certain impression materials require their use to be limited and be rinsed thoroughly immediately after application to avoid tissue irritation or material degradation. In another study, it was proved that chlorhexidine has sustained antimicrobial properties and lesser cytotoxicity in comparison to sodium hypochlorite; hence, it may be used safely for regular disinfection in prosthodontic practice as well [11]. Nonetheless, their effectiveness against particular microorganisms can be different; thus, an annual review of disinfection protocols in terms of clinical and microbiological findings may be appropriate.

Despite the meticulous design and execution of this study, several limitations should be acknowledged. First, the simulated microbial contamination might not perfectly replicate the complex and variable microbial environment of the oral cavity, potentially affecting the generalizability of the results. Additionally, the study was conducted in a controlled laboratory setting, which may not fully capture the variability encountered in clinical practice. The sample size, while statistically calculated, may still limit the ability to detect smaller differences between disinfection protocols. Furthermore, only two types of impression materials (alginate and PVS) were tested, which may not represent the full range of materials used in prosthodontic practice. Lastly, the study focused on short-term disinfection effects and did not assess the long-term stability and accuracy of the disinfected impressions, which could be an important factor in clinical applications. Future studies should address these limitations by including a broader range of impression materials, more diverse microbial conditions, and long-term evaluations to enhance the clinical relevance and applicability of the findings. In this regard, the present study is novel, as the previous study mostly focused on microbial contamination of dental impressions and its short-term effects. Long-term clinical follow-up studies concerning the sustainability or stability of this disinfection for long periods of time, like material deterioration, biofilm development, and patient-related judgments, are needed.

## Conclusions

In conclusion, this study provides valuable insights into the effectiveness of different disinfection protocols for dental impressions in prosthodontics. These findings underscore the importance of selecting an appropriate disinfection method based on its antimicrobial efficacy, biocompatibility, and impact on impression material properties. Clinicians should prioritize patient safety and infection control while maintaining the integrity and accuracy of dental impressions. Further research is needed to validate these findings, explore alternative disinfection methods, and address the remaining gaps in knowledge to advance evidence-based practices for prosthodontic infection control.
